# 

**Published:** 1886-11

**Authors:** 


Whole Number, 322 NOVEMBER, 1886. Vol. LIII. Number 5.

LIBRARY INDEXED S.G.O.

### THE CHICAGO MEDICAL JOURNAL AND EXAMINER

EDITORS:

S. J. JONES, M.D., LL.D. HAROLD N. MOYER, M.D.

PUBLISHED MONTHLY BY

THE CLARK & LONGLEY COMPANY,

Under direction of

THE CHICAGO MEDICAL PRESS ASSOCIATION,

308-316 Dearborn Street.

